# Assessing Gender and Sexual Harassment in Iranian Academia: Persian Adaptation and Validation of the Sexual Experiences Questionnaire

**DOI:** 10.7759/cureus.93667

**Published:** 2025-10-01

**Authors:** Amirmohammad Azizzadeh, Siavash Azimi Nasab Peyvasti, Mahasti Alizadeh, Shirin Ahmadnia

**Affiliations:** 1 Student Research Committee, Tabriz University of Medical Sciences, Tabriz, IRN; 2 Medical School, Tabriz University of Medical Sciences, Tabriz, IRN; 3 Medical Education Research Center, Health Management and Safety Promotion Research Institute, Department of Community Medicine, Faculty of Medicine, Tabriz University of Medical Sciences, Tabriz, IRN; 4 Faculty of Social Sciences, Allameh Tabataba’i University, Tehran, IRN

**Keywords:** gender-based violence, medical education, sexual harassment, survey questionnaires, validation study

## Abstract

Background

Sexual harassment remains a significant and widespread challenge in academic institutions worldwide. There is a significant lack of understanding and validated measurement tools for this issue in Iran. This study aimed to translate, culturally adapt, and validate the sexual experiences questionnaire for use among the Persian-speaking university student population.

Methods

This study developed a linguistically and culturally adapted Persian version of the shortened Sexual Experiences Questionnaire (p-SEQ). The translation followed established guidelines, involving forward and back-translation, expert panel reviews, and a pilot study with 36 students. A total of 441 students from a university in northwestern Iran were recruited using nonprobability sampling, with 391 completing the survey. Validity was assessed through face, content, and construct validity analyses, including exploratory and confirmatory factor analysis (EFA & CFA). Reliability was evaluated using Cronbach’s alpha and McDonald’s omega.

Results

The sample had a median age of 22 and 59.1% of respondents identified as female participants. EFA suggested a three-factor solution as the most interpretable, explaining 63.97% of the variance. However, CFA revealed that a modified four-factor model, aligning with the original domains of sexist gender harassment, crude gender harassment, unwanted sexual attention, and sexual coercion, provided the best fit (minimum discrepancy/degrees of freedom (CMIN/DF)=3.712, comparative fit index (CFI)=0.946, root mean square error of approximation (RMSEA)=0.083, standardized root mean square residual (SRMR)=0.048). The p-SEQ demonstrated strong internal consistency, with an overall Cronbach’s alpha of 0.880 and McDonald's omega of 0.858 and subscale alphas ranging from 0.842 to 0.935. The scale also showed good convergent and discriminant validity.

Conclusion

The p-SEQ is a psychometrically sound, valid, and reliable instrument for assessing sexual and gender harassment among Iranian university students. This tool addresses a critical gap in the existing literature and provides a psychometrically sound instrument in this single site sample. multi-site replication and invariance testing are needed before routine policy use within academic institutions in Iran.

## Introduction

Sexual harassment (SH) remains a widespread and persistent problem that goes beyond cultural, geographic, and institutional boundaries, impacting individuals in various environments such as workplaces, educational settings, and broader social contexts [1]. There are multiple definitions of SH across institutions and populations. The Equal Employment Opportunity Commission’s definition of SH consists of verbal or physical acts with sexual intent, unwelcome sexual advances, or requests for sexual favors. Workplace SH may lead to the creation of an intimidating or hostile work environment or become quid pro quo harassment, that is, a condition of employment or career advancement [1].

Academia, as a work environment, is not immune to occurrences of harassing behaviors. It has been reported that as many as 42% of university employees report experiencing harassing behaviors [2]. Students, as a population subset within the world of academia, are particularly at risk of experiencing harassment. Across the hierarchy of academic medicine, experiencing SH has been reported by 52% of medical students [3]. A meta-analysis of studies from various regions has also found that the global prevalence of SH among higher education students is approximately 36.9% [4].

The prevalence of SH in Iranian higher education is not well understood. Recent social movements in Iran, such as the MeToo movement, have increased public discourse on gender-based violence, though empirical data on sexual harassment in academia remain limited. To address issues related to the exploitation of power and authority, such as SH, understanding the true scope of the issue is crucial. This in turn emphasizes the value of standardized and validated measurement tools for assessing SH. One such tool is the Sexual Experiences Questionnaire (SEQ). Developed by Fitzgerald et al. [5], the SEQ is the most widely used tool for objective assessment of sexual and gender harassment behaviors endured by an individual. Several different adaptations of this measure are already available for different cultural and environmental contexts [5-12]. SEQ has been repeatedly validated in contexts where hierarchical structures, such as mentorship in an academic setting, are predominant.

In light of the scarcity of research on SH in Iran [4], particularly in Iranian universities, as well as the lack of a standardized tool to quantitatively evaluate SH among the Iranian and Persian-speaking populations, this study aimed to translate and culturally adapt the SEQ into Persian and determine its validity and reliability.

## Materials and methods

Instrument

The current study aimed to procure a culturally and linguistically adapted version of the SEQ for the Persian-speaking Iranian population. The SEQ is a valid and reliable questionnaire used for the quantitative assessment of gender and sexual harassment. The subscales of this tool are measured using behavior-based questions that center on the frequency of sexual and gender harassment, both physical and nonphysical, that an individual may experience over a given timeframe. The original survey consisted of four domains of sexist gender harassment, crude gender harassment, unwanted sexual attention, and sexual coercion.

The specific version of the SEQ used in this study was the shortened version of the Sexual Experiences Questionnaire-Department of Defense (SEQ-DoD) [8]. This shortened version is available for academic and non-commercial research, and its use in this study was in accordance with these terms. It was obtained through the Administrator-Researcher Campus Climate Consortium (ARC3) [13]. The shortened form was chosen as it minimized the participant burden while retaining the factor structure and essence of the original tool. This version has been incorporated into the ARC3 and Cultivating Learning and Safe Environments (CLASE) surveys [13,14]. The full list of items and their back-translations are available in the appendix.

Translation

The translation process followed the guidelines set forth by the World Health Organization [15]. Initially, a bilingual translator with expertise in public health translated the SEQ from English into Persian, resulting in a single forward translation. A panel of experts, consisting of sociologists, community medicine, and emergency medicine specialists, discussed the cultural and linguistic fitness of the forward-translated questionnaire, and revisions were made to enhance the comprehensibility and fluency of the questions. Additionally, the compliance of items with the Iranian prevention and mitigation of sexual harassment in scientific environments guideline was assessed, and example phrases were introduced to some items to align with the local culture and reduce confusion, while remaining loyal to the source material. Items one, two, three, six, and fourteen received local-example phrases (see appendix) as per the recommendations of the panel of experts. Pilot interviews did not indicate any increase in social desirability or cueing bias. Furthermore, similar example phrases have also been added to the previously published studies [13,14]. This version was then back-translated into English by an expert who was blinded to the original SEQ. The back-translated version was compared to the original SEQ to ensure conceptual equivalence. A final 10-person committee of experts in the fields of community medicine, nursing, ethics, psychology, and sociology reviewed the translation and made necessary recommendations. After implementing the necessary changes, an expert committee evaluated semantic equivalence and approved the final version after two rounds of revisions.

Pilot study

A pilot study with 36 university students was conducted to assess the initial reliability and concerns regarding questionnaire dissemination methods. The analysis revealed a Cronbach’s alpha of 0.864 for the pilot study. Interviews were conducted with participants to determine weaknesses and improve the overall survey.

Sampling method

The minimum sample size was determined as roughly 10-15 times the number of items in our questionnaire, which resulted in a minimum sample size of 160-240 [16]. A total of 441 participants were recruited from a university in northwestern Iran using non-probability sampling. Participants were recruited using a structured convenience sampling approach. To minimize selection bias, all eligible students were invited during scheduled class sessions across varied disciplines and academic years. Recruitment was also conducted across multiple faculties and classrooms using standardized announcements and online invitations. No incentives were offered, and participation was voluntary and anonymous. Inclusion criteria were: age ≥18 years, enrollment as a university student for at least one semester, fluency in Persian, and provision of informed consent. Participants were excluded if they did not consent to participate in the survey or anonymous publication of the results.

Face validity

Face validity was assessed through qualitative feedback from the pilot study group as well as the expert panels.

Content validity

An expert panel consisting of 10 specialists evaluated the content validity of the Persian SEQ (p-SEQ). They were asked to critically evaluate each question from a cultural and scientific standpoint and to provide their opinions. Each item was evaluated for relevance using a four-point scale of relevance, and both item-level content validity index (I-CVI) and scale-level content validity index (S-CVI/Average) were calculated. They also rated each question on a scale of ‘not necessary,’ ‘useful but not necessary,’ and ‘necessary,’ which was used to calculate the content validity ratio (CVR). Based on Lawshe’s table for 10 experts, CVR ≥0.62 indicates necessity at p<0.05. Items with a CVR of less than 0.62 warranted a second look and revision. The I-CVI values ranged from 0.70 to 1.00, with 15 out of 16 items exceeding the minimum threshold of 0.78, indicating strong expert agreement. The S-CVI/Average was 0.94, confirming strong overall content validity (Appendix). One item (question 12) fell below acceptable thresholds (I-CVI=0.70; CVR=0.60), suggesting limited consensus on its relevance and necessity. Upon review, this item was found to contain culturally ambiguous phrasing that may have led to inconsistent interpretation. To address this, the item was reworded to enhance clarity and contextual appropriateness based on expert feedback and pilot testing. The revised version was re-evaluated by the experts and showed improved clarity and alignment with the construct of sexual harassment. Given the strong performance of the remaining items and the proactive revision of the low-scoring item, the final version of the p-SEQ was deemed to have satisfactory content validity for use in the Iranian university context.

Construct validity

Construct validity was assessed using exploratory factor analysis (EFA) and confirmatory factor analysis (CFA). EFA was conducted to identify the underlying factor structure. The criteria for factor retention included eigenvalues larger than 1, factor loadings >0.4, and scree plot assessment. EFA was performed using a maximum-likelihood analysis with ProMax rotation. Sample size adequacy was determined by the Kaiser-Meyer-Olkin (KMO) test, where a value above 0.7 was considered adequate. Bartlett’s test was used to evaluate the correlation of the data. Models with two-, three-, and four-factor solutions were forced and compared with each other. All statistical analyses were conducted using IBM SPSS Statistics for Windows, Version 26 (Released 2019; IBM Corp., Armonk, New York, United States) and IBM SPSS AMOS Statistics for Windows, Version 24 (Released 2018; IBM Corp., Armonk, New York, United States) to confirm the identified factor structure, evaluate model fitness, and assess convergent and discriminant validity using CFA. CFA was conducted using maximum-likelihood estimation. Goodness of fit measures, including minimum discrepancy (CMIN), degrees of freedom (DF), CMIN/DF, root mean square error of approximation (RMSEA), comparative fit index (CFI), standardized root mean square residual (SRMR), and Akaike information criterion (AIC), were calculated. CMIN/DF<5, CFI≥0.9, RMSEA<0.08, and SRMR<0.08 were among the criteria used for assessing model fitness [17,18].

Criteria for establishing convergent validity included composite reliability (CR)>0.7, average variance extracted (AVE)>0.5, and CR>AVE [19]. The Fornell-Larcker criterion was used to establish discriminant validity, where the square root of the AVE of each factor should be greater than the correlation values with other factors [20].

Reliability assessment

Internal consistency of the p-SEQ was assessed using Cronbach’s alpha and McDonald’s omega. A Cronbach’s alpha value of 0.70 or higher was considered acceptable.

Statistical analysis

All statistical analyses were conducted using SPSS version 26.0 and SPSS AMOS version 24.0. The demographic characteristics of the participants were summarized using descriptive statistics. Missing data were handled by listwise deletion of incomplete entries.

Ethical considerations

Ethical and methodological approval was obtained from the institutional review board of Tabriz University of Medical Sciences prior to conducting the study (ID: IR.TBZMED.REC.1401.905). Informed consent was obtained from all participants for data collection and anonymized publication of the results. The services of a trained psychologist were provided to the participants. Information confidentiality, participant anonymity, and privacy were also ensured. Participant data were anonymized to the best of our ability and stored in encrypted storage.

## Results

Sample characteristics

**Figure 1:**
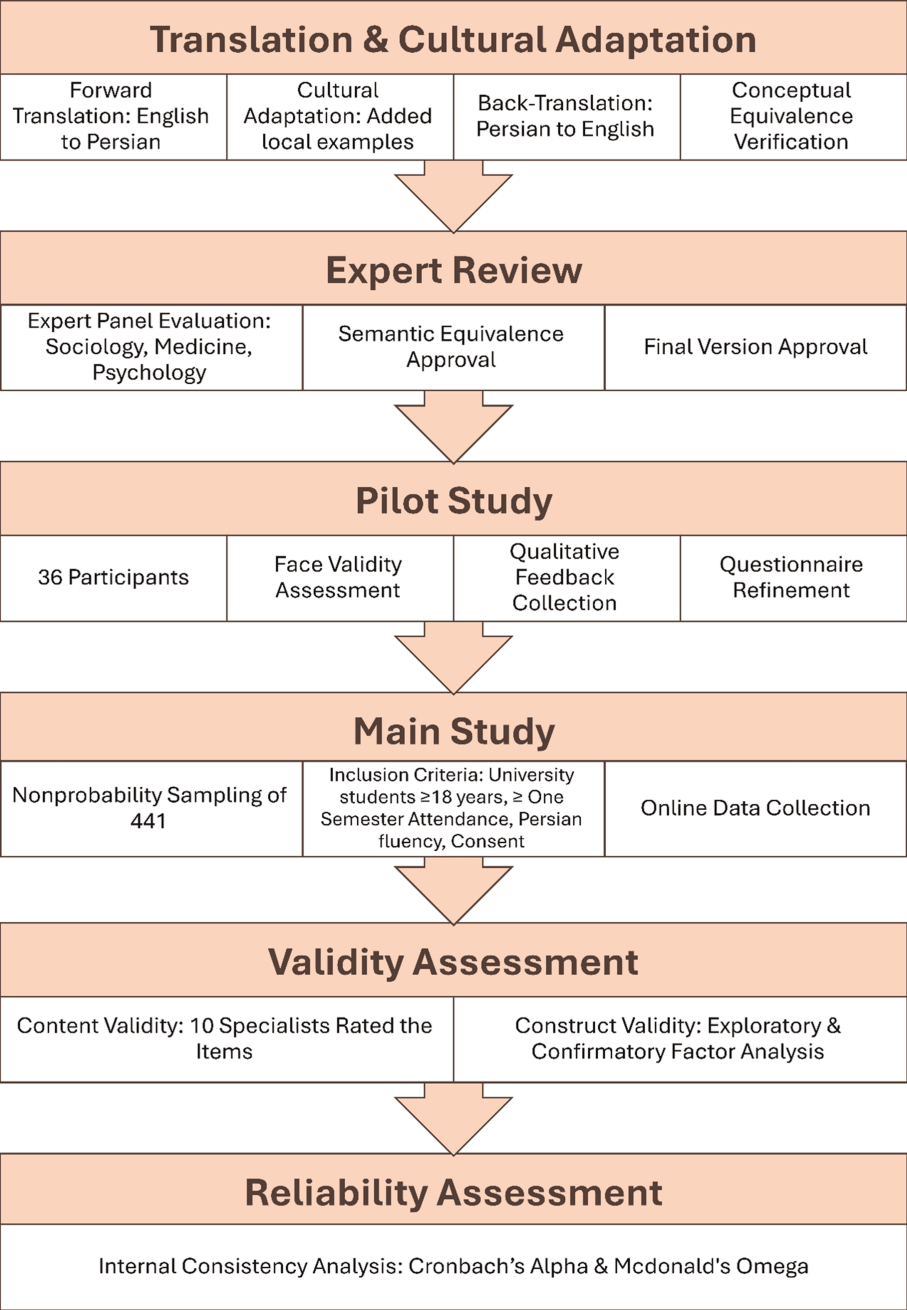
Adaptation flowchart of the Persian version of the sexual experiences questionnaire (p-SEQ)

A total of 441 participants were recruited, of which 391 completed questionnaires, resulting in a response rate of 88.7%. The median age was 22 years (IQR: 21-24). About 59.1% (n=231) of the respondents identified as female participants. The distribution of enrollment years was as follows: one year or less (26.6%), two years (16.1%), three years (17.6%), four years (16.1%), and five years or above (23.5%). Figure 1 shows the step-by-step flowchart of the study.

Exploratory factor analysis (EFA)

The KMO measure of sampling adequacy was 0.877, indicating that the data were suitable for the factor analysis. Bartlett’s Test of Sphericity was significant (χ² =4845.844, df=120, p<0.001), confirming the appropriateness of the data for the factor analysis. The EFA revealed a three-factor solution as the most interpretable structure with factor loadings above 0.6. The three-factor solution explained 63.97% of the variance in data. A two- and four-factor solution was forced, and each was able to explain 58.95% and 70.71% of the total variance, respectively. The factor structures of all three models were extracted for CFA.

Confirmatory factor analysis (CFA)

CFA was performed to validate the factor structure identified in the EFA. The four-factor model aligned with the original dimensions of sexual coercion, unwanted sexual attention, crude gender harassment, and sexist gender harassment. The four-factor model demonstrated acceptable fit indices (CMIN/DF=4.789, CFI=0.923, RMSEA=0.099, SRMR=0.056, and AIC=577.337). After the modifications, the fit indices improved further. The goodness-of-fit indices for the tested models are presented in Table 1.

**Table 1:** Goodness of fit indices from confirmatory factor analysis (CFA) of the tested models of the Persian version of sexual experiences questionnaire (p-SEQ) Statistical significance was set at p<0.05. CMIN: Chi-square statistic; DF: Degrees of freedom; p: p-value for chi-square test; CFI: Comparative fit index; RMSEA: Root mean square error of approximation; SRMR: Standardized root mean square residual; AIC: Akaike information criterion.

Model	CMIN	DF	p	CMIN/DF	CFI	RMSEA	SRMR	AIC
Two-factor model	1119.561	103	<0.001	10.87	0.788	0.159	0.08	1217.561
Three-factor model	879.477	101	<0.001	8.708	0.838	0.141	0.068	981.477
Three-factor model with modifications	419.029	94	<0.001	4.458	0.932	0.094	0.055	535.029
Four-factor model	469.337	98	<0.001	4.789	0.923	0.099	0.056	577.337
Four-factor model with modifications	356.39	96	<0.001	3.712	0.946	0.083	0.048	468.39

The four-factor model showed good convergent and discriminant validity. The CR values ranged from 0.850 to 0.936, and the AVE values ranged from 0.586 to 0.786, indicating adequate convergent validity. The Maximum Shared Variance (MSV) values were below the AVE values, supporting discriminant validity. The results are presented in Table 2.

**Table 2:** Convergent and discriminant validity metrics from the four-factor confirmatory factor analysis (CFA) models of the Persian version of sexual experiences questionnaire (p-SEQ) All correlation coefficients were significant at p<0.05. CR: Composite reliability; AVE: Average variance extracted; MSV: Maximum shared variance; MaxR(H): Maximum reliability (Heterotrait).

	CR	AVE	MSV	MaxR(H)	Sexual coercion	Unwanted sexual attention	Crude gender harassment	Sexist gender harassment
Sexual coercion	0.936	0.786	0.628	0.942	0.886			
Unwanted sexual attention	0.925	0.756	0.628	0.929	0.792	0.869		
Crude gender harassment	0.85	0.586	0.469	0.853	0.19	0.199	0.766	
Sexist gender harassment	0.872	0.631	0.469	0.879	0.214	0.245	0.685	0.794

**Figure 2:**
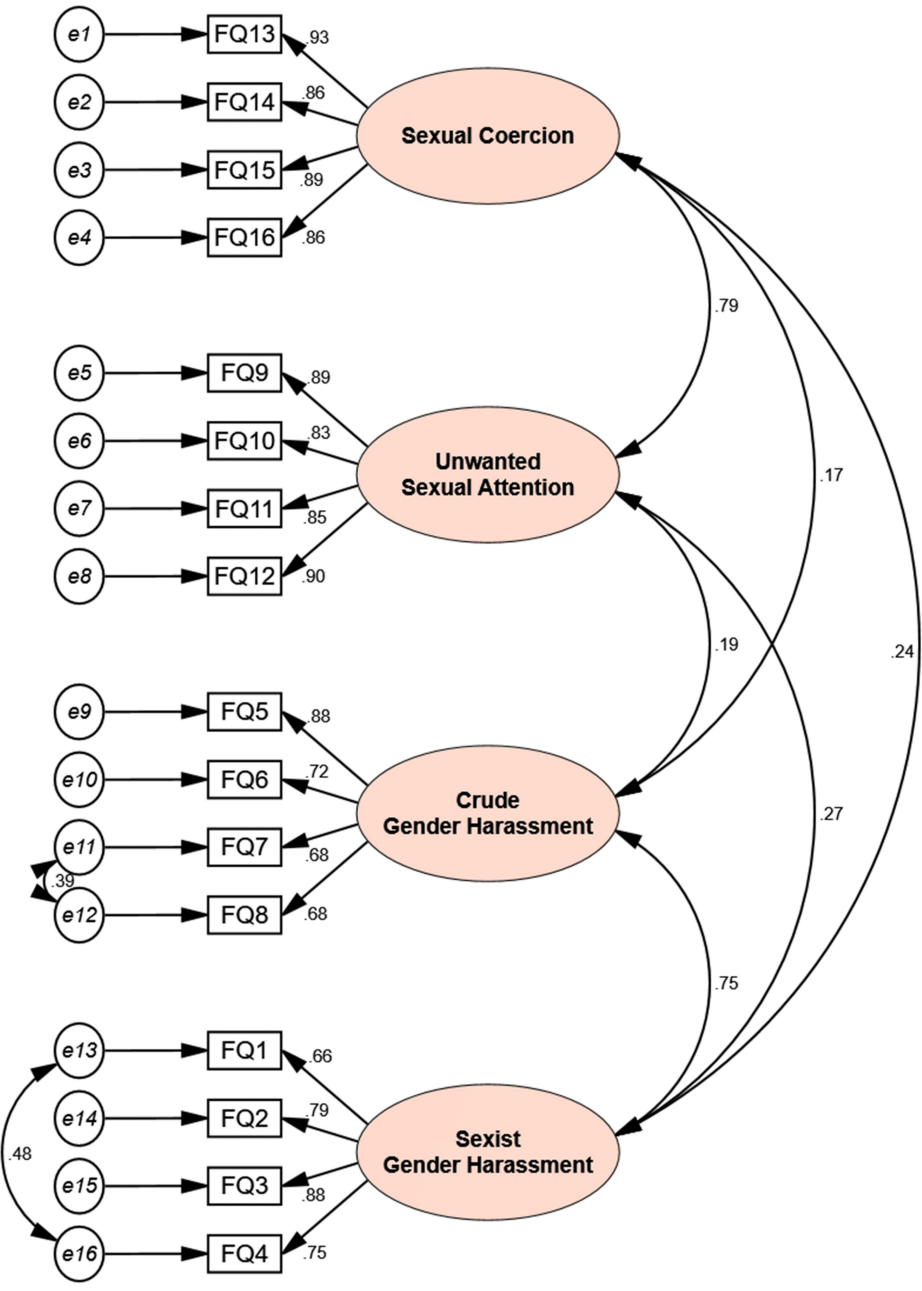
Structure of the modified four-factor model of the Persian version of the Sexual Experiences Questionnaire (p-SEQ) All displayed regression path coefficients were statistically significant at p<0.05.

The final modified four-factor model structure of the p-SEQ, which showed the best fit, is shown in Figure 2.

The model included the dimensions of sexual coercion, unwanted sexual attention, crude gender harassment, and sexist gender harassment.

Reliability analysis

Internal consistency of the p-SEQ was assessed using Cronbach’s alpha and McDonald’s omega. Cronbach’s alpha values for the subscales were as follows: crude gender harassment (α=0.868), sexist gender harassment (α=0.842), unwanted sexual attention (α=0.925), and sexual coercion (α=0.935). The overall Cronbach’s alpha for the questionnaire was 0.880. The McDonald’s omega values were also high, ranging from 0.848 to 0.935 for the subscales and 0.858 for the entire questionnaire, indicating strong internal consistency. The results are presented in Table 3.

**Table 3:** Internal consistency reliability of Persian version of sexual experiences questionnaire (p-SEQ) and its subscales as reported by Cronbach's alpha and McDonald's omega

Dimensions	Number of items	Cronbach's alpha	McDonald's omega
Crude gender harassment	4	0.868	0.871
Sexist gender harassment	4	0.842	0.848
Unwanted sexual attention	4	0.925	0.924
Sexual coercion	4	0.935	0.935
Entire questionnaire	16	0.880	0.858

## Discussion

This study translated, culturally adapted, and psychometrically validated the shortened version of the SEQ for use with Persian-speaking Iranian university students. The findings demonstrate that the p-SEQ is a psychometrically sound instrument with good reliability and validity for assessing the experiences of gender and sexual harassment in this population. The CFA supported a four-factor structure that aligns with the prior literature and the original domains of the SEQ-DoD. We termed these four subscales as sexist gender harassment, crude gender harassment, unwanted sexual attention, and sexual coercion.

The SEQ, developed by Fitzgerald et al., is the most widely utilized instrument in SH literature, which attempts to measure SH using a series of behaviorally oriented questions [5]. According to this model, SH is defined as the frequency of physical and non-physical harassing behaviors experienced over a given time period. Over time, several definitions of SH have emerged. However, the majority of definitions, including the one provided by the Iranian prevention and mitigation of sexual harassment in scientific environments guideline, appear to converge with the definition on which the SEQ is based. This original model of Fitzgerald et al. was further subdivided into three domains of gender harassment, unwanted sexual attention, and sexual coercion [5]. Subsequent research by Stark et al. aimed to reduce the original 23-item measure to 16 items while preserving its fundamental characteristics and maintaining comparable psychometric properties [8].

The Persian Women Workplace Culture (WWC) scale incorporated a subscale addressing SH within a broad organizational climate framework, although its application was primarily limited to non-academic work environments [21]. This SH subscale achieved a Cronbach's alpha of 0.7, which is suboptimal. Moreover, the SH subscale of this measure, comprising only two items, addresses only certain aspects of SH regarding unwanted sexual attention and advances [21]. Likewise, the nurse’s sexual harassment scale is a valid instrument for evaluating SH. This scale has demonstrated excellent internal consistency (Cronbach's alpha=0.94; Omega coefficient=0.94). Nevertheless, it is specifically designed for healthcare settings and fails to consider gender-related dimensions of harassment [22]. Among the earlier workers in the field of quantitative harassment research was the Iranian workplace sexual harassment survey, which emphasizes the theoretical framework of harassing practices. With 30 questions, this scale attempts to capture SH as two distinct categories of verbal and non-verbal harassment. This scale also encompasses items pertaining to sexual abuse, which must be differentiated from harassment. Moreover, the exact validity and psychometric properties of this scale remain unverified [23]. Another notable measure, the psychosexual harassment questionnaire, provided useful insights into physical, verbal, sexual, and virtual harassment. The questionnaire has demonstrated good reliability, with an overall Cronbach's alpha coefficient of 0.91, and reliability for the SH dimension was 0.88. Although this questionnaire included a sample of university students, the questions used for assessing SH did not incorporate elements of hierarchical power dynamics commonly observed in academia. Furthermore, the SH dimension of this instrument includes items pertaining to non-consensual sexual intercourse, which typically aligns with the category of sexual violence rather than SH [24]. These instruments collectively emphasize the need for a specialized, psychometrically sound tool that addresses the range of sexual and gender harassment in academia. Given that the SEQ has been demonstrated to be particularly well suited to environments characterized by organizational hierarchies, including academia, medicine, and the military, it was selected for adaptation to the Persian language.

The p-SEQ demonstrated a high internal consistency. The Cronbach's alpha coefficients were 0.868 for crude gender harassment, 0.842 for sexist gender harassment, 0.935 for unwanted sexual attention, and 0.925 for sexual coercion. The overall reliability of the scale was excellent, with a Cronbach’s alpha of 0.880. The reliability of the questionnaire was further supported by McDonald's omega values, which exceeded 0.8 for every subscale. The p-SEQ's fit indices and reliability coefficients were on par with or better than those found in earlier validation studies. The SEQ's original 23-item English version had a Cronbach's alpha of 0.94 overall, with subscale alphas ranging from 0.78 to 0.96 [8]. The current adaptation was based on the shortened version of the questionnaire, which showed subscale alphas ranging from 0.78 to 0.94 and an overall alpha of 0.92 [8]. This pattern of high reliability is consistent across various cultural and linguistic contexts. A recent 22-item adaptation of the SEQ to Jordanian Arabic reported an overall omega value of 0.98 [12]. Similarly, a Spanish validation study of the 23-item SEQ found an overall Cronbach's alpha of 0.934, with subscale values ranging from 0.803 to 0.899 [11]. Additionally, a total Cronbach's alpha of 0.95 was reported by the landmark ARC3 study, which used the shortened 16-item version of the SEQ [13]. The consistent demonstration of good reliability in different cultures and populations, which now includes the results of the current study, solidifies the SEQ's status as a reliable instrument for measuring sexual experiences.

Strengths and limitations

The primary strengths of this study include the rigorous, multi-stage translation and cultural adaptation process, the comprehensive assessment of various forms of validity (face, content, and construct) and reliability, and the large sample size. However, some limitations should be acknowledged. This study utilized a nonprobability sampling method and recruited participants from a single university in northwestern Iran. Institutional and cultural constraints precluded random sampling, as it would require access to personally identifiable student data, which was ethically and logistically unfeasible. While this sample represents a significant student population, the findings may not be generalizable to all university students or other populations within Iran. Future research should aim to validate the p-SEQ using more diverse samples across different universities and geographical regions in Iran. As SH can have detrimental effects on the victim's mental and physical health, researchers should explore the relationship between the experience of SH and health outcomes. Institutions can use the data to develop evidence-based training programs and confidential reporting systems to enhance student well-being and safety. As with most survey-based research, self-report bias and social desirability bias could have influenced the responses, although anonymity was assured to minimize this. Methodologically, our translation process utilized a single forward-translation followed by expert review, rather than a dual-translation design. Furthermore, while the addition of illustrative examples to some items was intended to increase clarity, this could have introduced a cueing effect, potentially narrowing participant interpretations of those questions. The cross-sectional design of the study did not allow for the assessment of test-retest reliability over a longer period or for causal inferences.

## Conclusions

In conclusion, the translation and validation of the short SEQ into Persian constitutes substantive advancement in the measurement of sexual and gender harassment in Iran. The scale’s robust factorial structure, high reliability coefficients, and sound validity support its adoption as a standard assessment tool for both research and practice. By addressing the limitations of previous instruments, p-SEQ fills a critical gap in the harassment measurement toolkit. The p-SEQ offers a standardized tool that can inform campus policies, support victim services, and guide institutional interventions to mitigate harassment and promote student safety.

## Appendices

**Table 4:** Persian version of the sexual experiences questionnaire (p-SEQ) and their corresponding items on the original SEQ

Original English SEQ item	Back-translated English from Persian items	Subscale
Treated you “differently” because of your sex?	Behaved differently toward you because of your gender? (e.g., mistreatment, insults, disrespect, or neglect)	Sexist gender harassment
Displayed, used, or distributed sexist or suggestive materials?	Showed or distributed sexist or "dirty" materials? (e.g., images, films, stories, or pornography that you found offensive)	
Made offensive sexist remarks?	Made unpleasant gender-related comments? (e.g., suggesting your gender is unsuitable for the type of work you do)	
Put you down or was condescending to you because of your sex?	Acted in a humiliating or discriminatory way toward you because of your gender?	
Repeatedly told sexual stories or jokes that were offensive to you?	Repeatedly told sexual stories and jokes that were offensive to you?	Crude gender harassment
Made unwelcome attempts to draw you into a discussion of sexual matters?	Made unwanted attempts to draw you into a discussion about sexual matters? (e.g., trying to discuss or comment on your sex life)	
Made offensive remarks about your appearance, body, or sexual activities?	Made offensive remarks about your appearance, body, or sexual activities?	
Made gestures or used body language of a sexual nature which embarrassed or offended you?	Used gestures or body language of a sexual nature that caused you embarrassment or distress?	
Made unwanted attempts to establish a romantic sexual relationship with you despite your efforts to discourage it?	Despite your discouraging actions, made unwanted attempts to create a romantic sexual relationship with you?	Unwanted sexual attention
Continued to ask you for dates, drinks, dinner, etc., even though you said “No”?	Despite your opposition, continuously requested to meet or invited you for dinner, etc.?	
Touched you in a way that made you feel uncomfortable?	Touched you in a way that made you feel uncomfortable?	
Made unwanted attempts to stroke, fondle, or kiss you?	Made unwanted attempts to caress or kiss you?	
Made you feel like you were being bribed with a reward to engage in sexual behavior?	By giving some kind of gift or special privilege for engaging in sexual behavior, made you feel like you were being bribed?	Sexual coercion
Made you feel threatened with some sort of retaliation for not being sexually cooperative?	By carrying out some kind of retaliatory behavior for your lack of sexual cooperation, caused you to feel threatened? (e.g., referencing an upcoming job/academic evaluation)	
Treated you badly for refusing to have sex?	Treated you badly because you refused to have a sexual relationship?	
Implied better treatment if you were sexually cooperative?	If you were sexually cooperative, their behavior toward you implicitly improved?	

**Table 5:** Expert ratings for Item-Level Content Validity Index (I-CVI) and Scale-Level Content Validity Index (S-CVI) Ten independent experts rated each item on a four-point scale for relevance which was the dichotomized as "1" = essential and "0" = not essential. Experts in Agreement: The number of experts (out of 10) who rated the item as essential.

Items	Expert 1	Expert 2	Expert 3	Expert 4	Expert 5	Expert 6	Expert 7	Expert 8	Expert 9	Expert 10	Experts in Agreement	I-CVI
1	0	1	1	1	1	1	1	1	1	1	9	0.9
2	1	1	1	1	1	1	1	1	1	1	10	1
3	1	1	1	1	0	1	1	1	1	1	9	0.9
4	1	1	1	1	1	1	0	1	1	1	9	0.9
5	1	1	1	1	1	1	1	1	1	1	9	0.9
6	1	1	1	1	1	1	1	1	1	1	9	0.9
7	1	1	1	1	1	1	1	0	1	1	9	0.9
8	1	1	1	1	1	1	1	1	1	1	10	1
9	1	1	1	1	1	1	1	0	1	1	9	0.9
10	1	1	1	1	1	1	1	1	1	1	10	1
11	1	1	1	1	1	1	1	1	1	1	10	1
12	0	0	1	1	1	1	1	0	1	1	7	0.7
13	1	1	1	1	1	1	1	1	1	1	10	1
14	1	1	1	1	0	1	1	1	1	1	9	0.9
15	1	1	1	1	1	1	1	1	1	1	10	1
16	1	1	1	1	1	1	1	1	1	1	10	1
Proportional Relevance	0.875	0.9375	1	1	0.875	1	0.9375	0.8125	1	1		S-CVI/average = 0.94
